# Cytotoxicity screening of *Thymus vulgaris* L. essential oil in brine shrimp nauplii and cancer cell lines

**DOI:** 10.1038/s41598-021-92679-x

**Published:** 2021-06-23

**Authors:** Haris Niksic, Fahir Becic, Emina Koric, Irma Gusic, Elma Omeragic, Samija Muratovic, Bojana Miladinovic, Kemal Duric

**Affiliations:** 1 Faculty of Pharmacy, University of Sarajevo, Zmaja od Bosne 8, 71000 Sarajevo, Bosnia and Herzegovina; 2 Faculty of Medicine, University of Nis, Dr Zoran Djindjic Boulevard 81, 18000 Niš, Serbia

**Keywords:** Cancer, Plant sciences

## Abstract

Among natural products, essential oils from aromatic plants have been reported to possess potent anticancer properties. In this work, we aimed to perform the cytotoxic concentration range screening and antiproliferative activity screening of chemically characterized *Thymus vulgaris* L. essential oil. In vivo bioassay was conducted using the brine shrimp lethality test (BSLT). In vitro evaluation of antiproliferative activity was carried out on three human tumor cell lines: breast adenocarcinoma MCF-7, lung carcinoma H460 and acute lymphoblastic leukemia MOLT-4 using MTT assay. Essential oil components thymol (36.7%), p-cymene (30.0%), γ-terpinene (9.0%) and carvacrol (3.6%) were identified by gas chromatography/mass spectrometry. Analyzed essential oil should be considered as toxic/highly toxic with LC_50_ 60.38 µg/mL in BSLT and moderate/weakly cytotoxic with IC_50_ range 52.65–228.78 µg/mL in vitro, according to evaluated cytotoxic criteria. Essential oil induced a dose-dependent inhibition of cell proliferation in all tested tumor cell lines and showed different sensitivity. Dose dependent toxicity observed in bioassay as well as the in vitro assay confirmed that brine shrimp lethality test is an adequate method for preliminary toxicity testing of *Thymus vulgaris* L. essential oil in tumor cell lines.

## Introduction

According to official data from the World Health Organization, cancer is the second leading cause of death in the world population and it is estimated that 9.6 million people died from this disease in 2018^1^. According to the latest issue of the International Agency for Research on Cancer (IARC), September 2018, lung cancer dominates, as the most common form of cancer and is also the most common cause of death among men. It is followed by prostate cancer and colorectal cancers by incidence and liver and stomach cancers by mortality^2^. When it comes to women, breast cancer is the most commonly diagnosed cancer and is the leading cause of cancer mortality, followed by colorectal and lung cancer by incidence. Cervical cancer ranks fourth in incidence and mortality^2^. Acute lymphoblastic leukemia is the most common hematological cancer in children and adolescents, representing 20% of all cancers diagnosed in persons aged < 20 years in the United States^3^.

More than two thirds of drugs currently used as anticancer agents have plant origin. Key hallmarks of anticancer therapy approach include inducing cancers cell death and reducing their sustained proliferative signaling and growth^4^.

Certain essential oils (EOs) have been labeled as promising anticancer agents and are currently being investigated for their cytotoxic and antiproliferative activities in cancer cell lines or experimental animals^5^. Different mechanisms have been reported for cytotoxic effects of EOs or their constituents. These include induction of cell death by apoptosis and/or necrosis, antimutagenic, antiproliferative, antioxidant properties, cell cycle arrest, and loss of key organelle functions^6^. According to the research findings, essential oils possess anticancer potential against mouth, breast, lung, prostate, liver, colon, and brain cancer and even in leukemia^7^. Studies indicate that the specific components of essential oils increase the cytotoxic activity of chemotherapeutic drugs (docetaxel, paclitaxel, 5-fluorouracil) on different cell lines and thus open the possibility of reducing their dose while providing the same effect^8^.

Oxygenated monoterpenes and monoterpene hydrocarbons represent the main chemical components of *Thymus vulgaris* L. essential oil. The natural terpenoid thymol and its phenol isomer carvacrol are the most abundant compounds of this oil. Non-clinical data reported that thyme essential oil exhibits strong antibacterial, antifungal, spasmolytic, antioxidant, anti-inflammatory activities^9^. On the basis of a summary review of available data, it is evident that thymol may be a promising plant-derived cancer chemotherapeutic agent^10^. *T. vulgaris* L. extracts are reported to possess anticancer potential, according to the several researches. These include significant free radical scavenging activity and proapoptotic effects in the human BC T47D cell line^11^, inhibition of proliferation of colorectal HCT116 cancer cell line^12^, and tumor inhibitory effects on human leukemia THP-1 cells^13^. The capability of the *T. vulgaris* L. essential oil to significantly inhibit the growth of human oral cavity squamous cell carcinoma has been reported^14^. Evaluation of cytotoxic activity of thyme essential oil demonstrated antiproliferative and proapoptotic effects on the two independent human breast adenocarcinoma cell lines^15^. However, a further investigation is needed in order to estimate the anticancer potential of thyme essential oil.

It has been proven that the brine shrimp lethality test (BSLT) has a good correlation with cytotoxic activity in some human solid tumors^16^. Application of BSLT in cytotoxic assays has been described for several essential oils^17^. Although the BSLT is able to identify strong anticancer activity of tested compound, its main limitation is its sensitivity to distinguish between strong to moderate and weak anti-cancer potentials^18^. Therefore, the BSLT represents a screening tool for potential cytotoxins, but a more sensitive distinction of anticancer activity requires testing on human cancer cells.

This paper focuses on the analysis of the antiproliferative activity of the chemically characterized *T. vulgaris* L. essential oil against breast adenocarcinoma (MCF-7) cell line, lung carcinoma (H460) cell line and acute lymphoblastic leukemia (MOLT-4) cell line. Moreover, cytotoxic concentration range screening and cytotoxicity validation in cancer cell lines and brine shrimp lethality of thyme essential oil were tested and evaluated.

## Results

### Chemical composition analysis

The essential oil of *T. vulgaris* L. isolated by hydro-distillation was of yellowish color, clear with an aromatic odor of thymol, in total yield of 1.5% (v/w) on dry weight basis. Qualitative and quantitative analytical results were obtained using gas chromatography/mass spectrometry (GC–MS) (see Supplementary Fig. S1). Table 1 shows the compounds identified in the essential oil of *T. vulgaris* L. in order of elution on ZB-5 MS capillary column. The percentage content of the individual components, retention indices and chemical class distribution are summarized.

**Table 1:** Chemical composition of *Thymus vulgaris* L. essential oil.

Component no	Components^a^	RT (minutes)	RI^b^	RI^c^	Percentage (%)
1	Methyl isobutyrate	2.77	780	780	0.40
2	α-Thujene	5.25	930	930	1.00
3	α-Pinene	5.45	932	934	1.50
4	Camphene	5.86	946	956	1.00
5	β-Pinene	6.53	974	978	0.70
6	1-Octen-3-ol	6.53	979	981	0.70
7	3-Octanone	6. 67	983	986	0.10
8	Myrcene	6.78	987	990	1.50
9	3-Octanol	6.99	988	991	0.10
10	α-Terpinene	7.60	1014	1020	1.90
11	p-Cymene	7.85	1024	1029	30.00
12	Limonene	7.97	1024	1033	0.70
13	1,8-Cineole	8.07	1026	1037	1.40
14	γ-Terpinene	8.83	1059	1062	9.00
15	Linalool	10.12	1096	1100	2.40
16	Camphor	11.73	1146	1152	0.70
17	Borneol	12.56	1165	1167	1.70
18	Terpinen-4-ol	12.83	1147	1183	0.90
19	α-Terpineol	13.32	1186	1196	0.40
20	Methyl thymyl ether	14.43	1235	1231	0.30
21	Methyl carvacryl ether	14.74	1244	1241	0.40
22	Bornyl acetate	16.32	1287	1286	0.30
23	Thymol	16.54	1290	1292	36.70
24	Carvacrol	16.78	1299	1298	3.60
25	β-Caryophyllene	20.71	1417	1420	0.40
	Total identified				100
	Monoterpene hydrocarbons				47.60
	Oxygenated monoterpenes				49.20
	Other monoterpenes				1.00
	Aliphatic compounds				1.30
	Sesquiterpene hydrocarbons				0.40
	Oxygenated sesquiterpene				0.50

^a^Compounds listed in order of eluation from a ZB-5 MS column.

^b^Literature retention indices.

^c^Indices relative to C8-C20 n-alkanes on the ZB-5 MS column.

Out of a total of 32 identified components, 25 components presented in Table 1 represent 97.8% of the total content of the thyme essential oil. This oil was characterized by high percentage of monoterpene hydrocarbons (47.6%) and oxygenated monoterpenes (49.2%), represented in almost equal quantities, and made up ~ 97% of the oil composition. In contrast, aliphatic compounds (1.30%), sesquiterpene hydrocarbons (0.4%) and oxygenated sesquiterpene (0.5%) fractions were lower. As shown in Table 1, p-cymene (30.0%) and γ-terpinene (9.0%) were the major components within the monoterpene hydrocarbon fraction. The most significant components within the oxygeneted monoterpene fraction were thymol (36.7%) and carvacrol (3.6%).

### Brine shrimp lethality test

The LC_50_ values obtained from brine shrimp lethality bioassay for *T. vulgaris* L. essential oil and thymol as positive control were 60.38 μg/mL and 16.67 μg/mL, respectively. The valorized toxicity of essential oil by comparison to Meyer’s and Clarkson’s toxicity index is summarized in Table 2.

**Table 2:** The results of cytotoxic activity of *Thymus vulgaris* L. essential oil and positive control thymol.

Compound	Concentration (µg/mL)		LC_50_ (µg/mL)	95% confidence interval		Toxicity class (Meyer/Clarkson)
*Thymus vulgaris* L. essential oil	[3.9–1000]		[60.38]	[27.12–134.26]		Toxic/highly toxic
Thymol	[3.9–1000]		[16.67]	[5.20–56.29]		Toxic/highly toxic

### Antiproliferative evaluation

**Figure 1:**
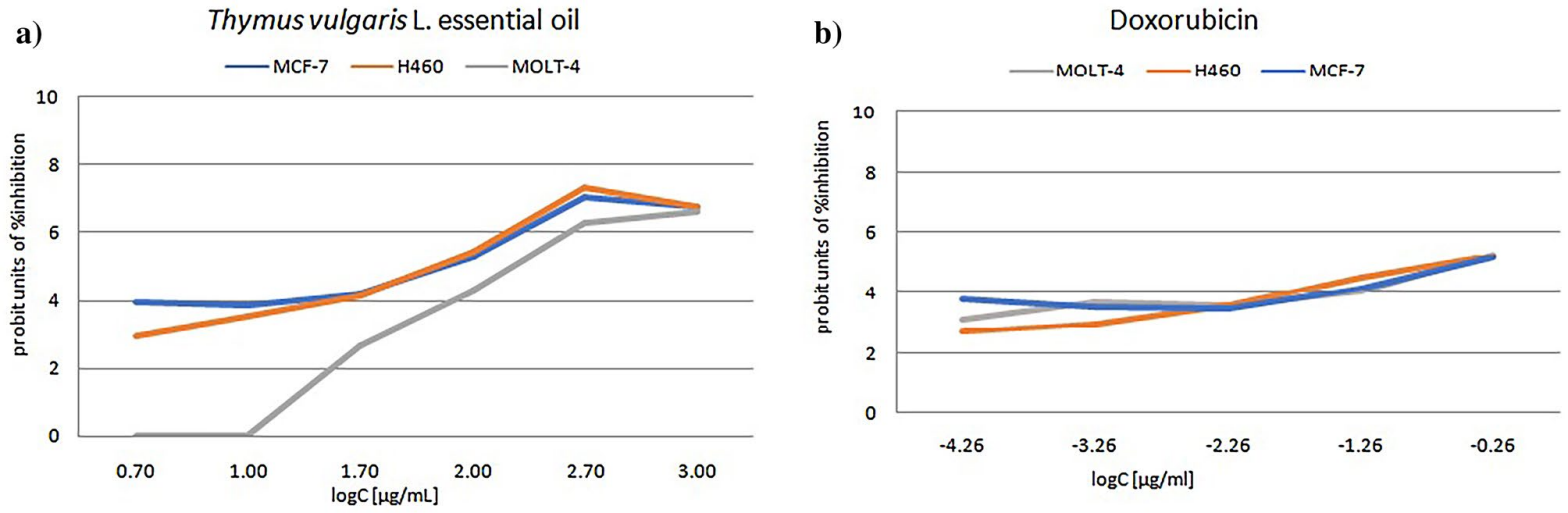
Probit units of growth inhibition of cancer cell lines. (**a**) Probit units of growth inhibition of MCF-7, H460 and MOLT-4 cells in vitro, assessed by MTT assay 72 h after the addition of *Thymus vulgaris* L*.* essential oil. (**b**) Probit units of growth inhibition of MOLT-4, H460 and MCF-7 cells in vitro, assessed by MTT assay 72 h after the addition of doxorubicin.

Results of the antiproliferative evaluation of tested essential oil and of the doxorubicin, as referent drug are presented by (Fig. 1a,b, 2a,b). Essential oil of the *T. vulgaris* L. at 72 h after treatment had inhibitory potential on proliferation of tested cancer cells. The results of the antiproliferative activity suggested that *T. vulgaris* L. essential oil induced a dose-dependent inhibition of cell proliferation in all tested tumor cell lines, although each culture showed different sensitivity, in accordance to determined IC_50_.

**Figure 2:**
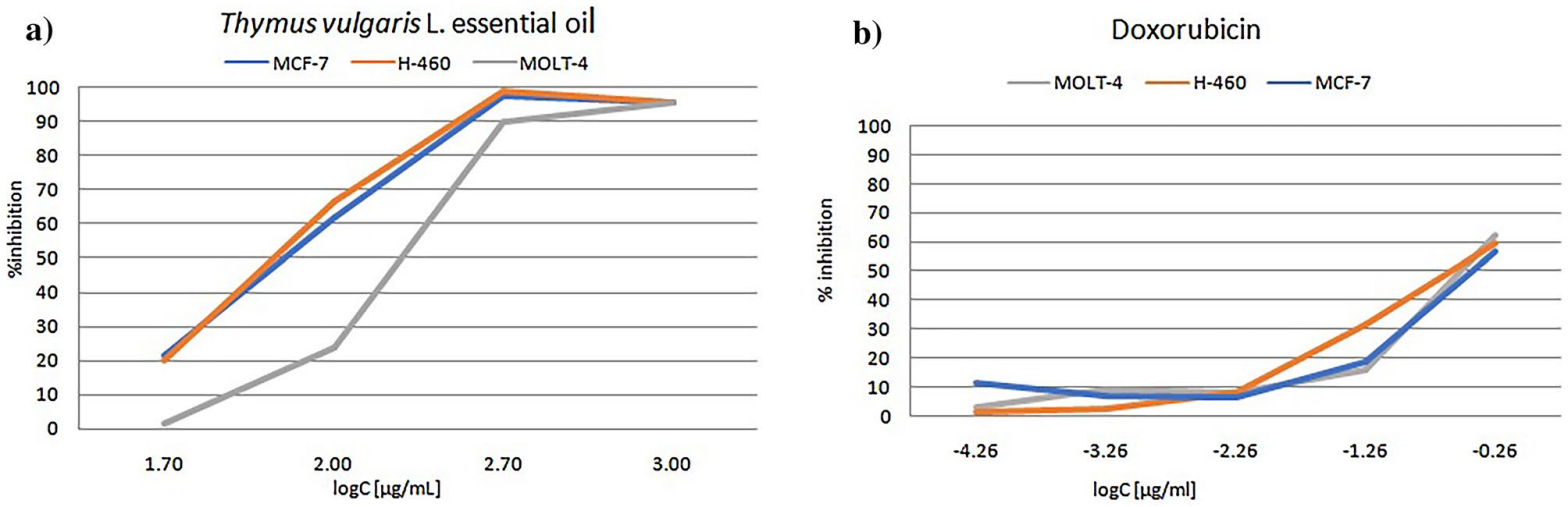
Growth inhibition of cancer cell lines. (**a**) Growth inhibition of MCF-7, H460 and MOLT-4 cells in vitro, assessed by MTT assay 72 h after the addition of *Thymus vulgaris* L*.* essential oil. (**b**) Growth inhibition of MOLT-4, H460 and MCF-7 cells in vitro, assessed by MTT assay 72 h after the addition of doxorubicin.

Table 3 presents the IC_50_ values determined from the dose–response curves of the thyme essential oil and the used anticancer drug on MOLT-4, MCF-7 and H460 cells lines. The strongest antiproliferative activity of *T. vulgaris* L. essential oil was observed against MCF-7 cell line (IC_50_ 52.65 μg/mL) followed by H460 cell line (IC_50_ 68.59 μg/mL) and MOLT-4 cells (IC_50_ 228.78 μg/mL).

**Table 3:** In vitro screening cytotoxic activity of *Thymus vulgaris* L. essential oil and positive control doxorubicin.

IC_50_ (µg/mL)						
Compound	Cell lines					
	MOLT-4	95% confidence interval	MCF-7	95% confidence interval	H460	95% confidence interval
*Thymus vulgaris* L. essential oil	[228.78]	[118.23–442.66]	[52.65]	[11.35–244.13]	[68.59]	[22.49–209.09]
Doxorubicin	[0.399]	[0.295–0.504]	[0.459]	[0.455–0.463]	[0.381]	[0.377–0.386]

IC_50_: the concentration that causes 50% growth inhibition, as assessed by MTT assay, 72 h after the addition of the test substances. The results are shown as mean values of quadruplicates**.**

## Discussion

Dried Thymi herba contains up to 2.5% essential oil^9^, which corresponds to the total yield of essential oil obtained in our study (1.5%) (v/w). According to the literature, different chemotypes of *T. vulgaris* L. have been reported, providing evidence of intraspecific chemical variability of this plant species. There are at least 6 chemotypes of *T. vulgaris* L.^19^, with different compositions of the essential oil. The main components are thymol, carvacrol, p-cymene, γ-terpinene, linalool, β- myrcene and terpinen-4-ol^9^. The above results show that chemical composition of the *T. vulgaris* L. essential oil, native to Bosnia and Herzegovina, was dominated by thymol (36.7%), p-cymene (30.0%), γ-terpinene (9.0%), carvacrol (3.6%). These results are in agreement with previous published data on thyme essential oil from different geographical regions in Europe^20^, reporting that monoterpene alcohol and monoterpene hydrocarbon fractions were the most abundant constituents in the oil of *T. vulgaris* L. The sum of phenolic compounds (thymol and carvacrol) in the studied oils varied from 19.4 to 84.4%, and the sum of their precursors (p-cymene and γ-terpinene) ranged from 5.7 to 38.5%^21^. The effect of different geographical zone on percentage composition was also confirmed by the research of Satyal et al. who worked on thyme collected from different regions^22^. The result of their study showed that the *T. vulgaris* L. collected from France showed higher percentage of linalool (76.2%) and linalyl acetate (14.3%) where the thyme obtained from Serbia showed the higher presence of geraniol (59.8%) and geranyl acetate (16.7%). The chemical composition analysis obtained suggests that *T. vulgaris* L. essential oil isolated in this study can be classified as ‘thymol’ chemotype, with thymol as the predominant compound, which complies with the definition in the European Pharmacopoeia^23^. Sum of the contents of thymol and carvacrol also meets the requirements of European Pharmacopoeia (minimum 40.0%)^23^.

The variations during the vegetative cycle in yield and percentage of chemical composition content is also reported^20^. Hudaib et el. confirmed that young plant, collected in June/July just before the end of the vegetative cycle, provided the best oil yield with also the highest % content of the monoterpene fractions (thymol and carvacrol)^20^. In this study, the appropriate harvest time of thyme material is chosen in order to obtain an essential oil with of appropriate quality and quantity.

The significant correlation between the BSLT and in vitro growth inhibition of human solid tumor cell lines demonstrated by the National Cancer Institute (NCI, USA) is significant because it shows the value of this bioassay as a pre-screening tool for antitumor drug research^24^. Hence, we assumed that this method could be used in evaluating essential oil toxicity for predicting cytotoxic concentration range and cytotoxicity validation in cancer cell lines. The toxicity of tested essential oil by comparison to Meyer’s and Clarkson’s toxicity index showed that analyzed essential oil should be considered as toxic/highly toxic with LC_50_ 60.38 μg/mL in BSLT. *T. vulgaris* L. essential oil showed 100% toxicity in brine shrimp model assay provided by Bogavac et al.^25^. Although the essential oil is obtained from the same plant species (*T. vulgaris* L.) the difference in LC_50_ values and % toxicity of the thyme essential oil exists, compared to our study. By comparing the GC–MS analysis of the tested oils, a difference in proportions of monoterpene alcohol and monoterpene hydrocarbon fractions is observed. In contrast to our results, the contribution of monoterpene alcohols (thymol and carvacrol), in EO obtained from Bogavac et al.^25^, is doubled, whereas the contribution of monoterpene hydrocarbons (p-cymene and γ-terpinene) is significantly decreased. The toxicity effect in BSLT of above mentioned EOs can be assumed, regarding the high percentage of thymol and carvacrol. Differences in the LC_50_ values of essential oils from different species of the genus Thymus in BSLT has also been evaluated. *Thymus serpyllum* L. essential oil showed LC_50_ value of 37.99 µg/mL^17^, compared to higher LC_50_ values (60.38 µg/mL) of *T. vulgaris* L. essential oil in our study. Toxicity of different extracts of *T. vulgaris* L. is also reported^26^. Al-Balushi et al. concluded that non polar organic extracts (chloroform and petroleum ether extracts) are toxic against the brine shrimp nauplii, whereas polar fractions are not showing toxic activity (hydroalcoholic extract)^26^. All in all, the thyme essential oil showed activity in the brine shrimp assay with LC_50_ values as relevant preliminary toxicity parameter for further toxicity studies.

In order to gain relevant data on cytotoxicity concentration range of thyme essential oil, it was necessary that in vitro testing was involved. Until now, no study had been conducted testing antiproliferative activity of thyme essential oil on lung carcinoma cell line H460 and acute lymphoblastic leukemia cell line MOLT-4. Our research contributes mainly to defining the IC_50_ values of tested essential oil for listed cell lines.

Our data demonstrated that *T. vulgaris* L. essential oil inhibited the viability of MCF-7, H460 and MOLT-4 cell lines in a concentration-dependent manner. At the concentrations ranging from 5 to 1000 μg/mL an antiproliferative effect was observed for each cell line MCF-7, H460 and MOLT-4 with IC_50_ values 52.65 µg/mL, 68.59 µg/mL, 228.78 µg/mL, respectively. Based on the results of the antiproliferative effect of thyme essential oil on the observed cell lines presented in (Fig. 1a,b), it is obvious that solid tumor cell lines MCF-7 and H460 showed very similar sensitivity to the essential oil. The hematological tumor cell line MOLT-4 is less sensitive to essential oil than solid tumor cell lines MCF-7 and H460, which correlates with the obtained IC_50_ values. Our research with *T. vulgaris* L. essential oil, native to Bosnia and Herzegovina with its unique chemical composition, contributes to previously published statements on good correlation between BSLT bioassay and cytotoxic activity in human solid tumors^16^. Although, there is a difference in sensitivity of solid and hematological tumor cell line to thyme essential oil, it can be noticed that plateau effect of percentage of inhibition of cell proliferation is observed in concentrations of 500–1000 µg/mL. For all tested cell lines, a high level of inhibition of cell proliferation in the indicated concentrations was approximately 96%. *T. vulgaris* L. essential oil showed moderate cytotoxic activity against MCF-7 and H460 cell lines, compared to weak cytotoxic activity against MOLT-4 cell line, according to a criteria based on NCI and Geran protocols^27^. Beside our research results, *T. vulgaris* L. essential oil has been observed to significantly inhibit growth of human oral cavity squamous cell carcinoma with IC_50_ value of 369.55 µg/mL^14^. Cytotoxicity of thyme essential oil towards head and neck squamous cell lines (HNSCC) was evaluated by standard 2,3-bis [2-methoxy-4-nitro-5-sulfophenyl]-2H-tetrazolium-5-carboxanilide inner salt (XTT) assay, after 72 h incubation. In vitro study performed by Kubatka et al. suggested the proapoptotic potential of *T. vulgaris* L. essential oil, by showing the activation of a mitochondria-induced apoptosis pathway in two independent human breast adenocarcinoma cell lines (MCF-7 and MDA-MB-231)^15^. All these studies, were conducted with *T. vulgaris* L. essential oil of similar chemical composition to the one used in this study.

Given the limited data on an in vitro antiproliferative activity of thyme essential oil, the obtained IC_50_ values for the tested tumor cell lines represent a significant contribution to the database on a cytotoxic activity of essential oils. In some cases, this activity was attributed to specific components of the oil. The chemical complexity of the essential oils as a phyto-complex contributes to its biological effects, as each constituent takes part in the overall outcome and may modulate the effects of the others. Studies on individual ingredients might report results that do not recapitulate the effect of treatment with the phyto-complex as a whole.

Our GC–MS analysis showed that the most abundant metabolites in thyme essential oil are thymol and p-cymene. Based on previously reported in vivo and in vitro results, both molecules could be possible candidates for lead molecules of cytotoxic activity^14,15^. However, the cytotoxic effect of other components present at lower percentages in the essential oil must be considered. From the early reports, thymol has been reported to exert anti-carcinogenic activity through a different mechanism of action on the cancer cells lines. These include oxidative stress and cancer cell death^28^, apoptotic cancer cell death^29^ and antiproliferative effects on cancer cells^30^. Contrary, antioxidant activity^31^, protective effects^32^, antiapoptotic effects^31^, anti-inflammatory/immunomodulatory effects^33^ and antigenotoxic effects^34^ may be the key mechanisms of thymol’s anti-carcinogenic activity in normal cells. The interesting observation reported by Oliviero et al.^35^ was that the same treatments with thyme extract that had no effect on viability of normal human epithelial cells, were able to induce necrotic cell death in H460 lung cancer cell line. The growth of A17 triple negative breast carcinoma cells transplanted into Friend leukemia virus B (FVB) syngeneic mice was remarkably inhibited by the ruthenium (II) p-cymen complex throught inhibition of the tumor infiltration of regulatory T cells^36^. The induction of p53 protein expression in MCF-7 cells and reduction of their ability to invade have also been reported for ruthenium (II) p-cymene complexes^37^. Ferraz et al. demonstrated that p-cymene was cytotoxic to mouse melanoma B16-F10 cell lines, showing IC_50_ = 20.06 µg/mL^38^. Carvacrol induced apoptosis via mitochondrial membrane permeabilization in the metastatic breast cancer cell line (MDA-MB-231)^39^. These data, together with the results obtained by our group, demonstrate that thymol, p-cymene and *T. vulgaris* L. essential oil as a phyto-complex have a potential therapeutic significance and should not be overlooked as possible therapeutics in treating cancer. The benefit of thyme essential oil as a chemotherapeutic of plant origin is reflected in the different mechanism of action on normal cells compared to cancer cells^40^. During the use of thyme oil expectorant, spasmolytic, antioxidant, antimicrobial, invigorating, eupeptic, appetizing and choleretic properties have been manifested^9^. When administered in the recommended posology, *Thymus vulgaris* L. essential oil is recognized as safe^9^. The Food and Drug Administration (FDA) has stated for thyme oil (for which thymol is a component) that it is Generally Recognized as Safe (GRAS) essential oil^41^.

## Conclusions

Dose dependent toxicity was observed in brine shrimp lethality assay as well as the in vitro assay conducted in cancer cell lines supporting the presence of bioactive compounds in *T. vulgaris* L. essential oil. The results of our study show that the essential oil of *T. vulgaris* L. has antiproliferative activity against several malignant cell lines (MOLT-4, MCF-7 and H460). This study has, for the first time, demonstrated cytotoxic activity of thyme essential oil to a lung carcinoma and an acute lymphoblastic leukemia cell line. We suggest that the antiproliferative activity of phytochemicals present in *T. vulgaris* L. essential oil is cancer cell line dependent. Moreover, we assumed that results obtained in this study point to the activity of the more abundant compounds thymol and p-cymene in analyzed thyme oil. The previously stated positive correlation between BSLT and in vitro testing on cancer cell lines are in accordance with results obtained in this study. This confirms, that BSLT is an adequate method for preliminary toxicity testing in tumor cell lines.

## Methods

### Chemical and materials

#### Plant material

Aerial parts of wild growing plant, *Thymus vulgaris* L., in the first year of growth, were collected in June 2018 in Bosnia and Herzegovina (location: Višići, near the city Čapljina, Latitude N 43° 04′ 02″; Longitude E 17° 42′ 44″). The permission for collecting *Thymus vulgaris* L. was obtained by the Federal Ministry of Enviroment and Tourism, Bosnia and Herzegovina. The identification of the experimental plant material,Voucher specimen No. 115/18, was carried out by the plant taxonomist Dr. Samir Đug, and voucher specimen deposited in the official herbarium of the Department of Biology and Ecology, Faculty of Sciences, University of Sarajevo. After plant material (1 kg) had been collected, the samples were dried at room temperature. Plant material was stored under nitrogen.

### Isolation of the essential oil

A total of 30 g air-dried herb (leaf, flower and stem) of *T. vulgaris* L. was mixed with 400 mL of distilled water. The mixture was subjected to hydro distillation using a Clevenger-type apparatus (Klaus Hofmann GmbH, Germany) according to the European Pharmacopoeia 8.0. at flow rate of 2.5 ml/min for 2 h^23^. The essential oil was collected, determined volumetrically, dried under anhydrous sodium sulphate and stored in a refrigerator at 4 °C in a vial prior to analysis. Distillations were performed at least three times and the mean content of essential oil was calculated.

### Gas chromatography-mass spectrometry (GC–MS) analysis of the essential oil

The chemical components of the analyzed essential oil were separated by the gas chromatography and identified by the mass spectrometry using Thermo Scientific GC–MS system (DSQ II GC/MS with Trace GC Ultra, Palo Alto, USA). Samples were analyzed on the capillary column ZB-5 MS 30 m × 0.25 mm, film thickness 0.2 μm, (Phenomenex, Torrance, USA). GC–MS settings were as follows: the initial oven temperature was held at 60 °C for 1 min and ramped at 4 °C/min rising to 250 °C; helium was used as carrier gas at a flow rate of 1 mL/min; the sample (0.1 μL) was injected manually at the split/splitless injector temperature of 260 °C, with a split ratio 1:50 and transfer line temperature was set to 270 °C for GC–MS analyses. Mass spectra were obtained at 70 eV (EI), the scan range was 45–350 *m/z* and Xcalibur version 2.0.7. was used for result processing and quantification. The components of the essential oil were identified by obtained GC–MS spectra and retention indices (RI) relative to C8-C20 n-alkanes. AMDIS computer program version 2.62 was used for GC–MS data processing using NIST library version 2.0. Spectra and obtained retention indices were compared with data already available in the literature^42^.

### Brine shrimp lethality test (BSLT)

The brine shrimp lethality test was performed according to the protocol of Asaduzzaman et al.^43^. The method estimates in vivo lethality in a simple zoological organism, using nauplii of the brine shrimp *Artemia salina.*

Artificial sea water (ASW) was prepared by mixing 27 g commercially available salt mixture (Dohse Aquaristik GmbH & CO. KG, Germany) with 900 mL of distilled water, per instructions given. *Artemia salina* eggs (Dohse Aquaristik GmbH & CO. KG, Germany) were added in the small commercial tank for nauplii hatching and incubated in ASW under a halogen lamp, providing direct light and warmth. Twenty-four hours were allowed to hatch the shrimp and to be matured as nauplii. The hatched shrimps are attracted to the light and nauplii free from brine shrimp eggs were collected from the illuminated compartment of the tank. Ten nauplii were counted macroscopically in the stem of the graduated Pasteur pipette against a lighted background and transferred to test tubes. By dissolving the essential oil sample in a convenient solvent (DMSO), a stock solution is made (2000 µg/mL), which was used for serial dilutions to prepare the concentrations 1000; 500; 250; 125; 62.5; 31.25; 15,625; 7.81; 3.9 µg/mL. Each test tube contained essential oil sample dilution in DMSO (2.5 mL) and commercial salt mixture with 10 brine shrimp nauplii (2.5 mL). DMSO and thymol were used as negative and positive controls, respectively. The test was conducted with three replicates for each treatment and ten nauplii per replicate. Each test tube was maintained under illumination during 24 h. Survivors were counted macroscopically, by two independent counters.

### Antiproliferative experiments

The antiproliferative effect of the *T. vulgaris* L. essential oil was determined using MTT assay^44^. In vitro investigation of antiproliferative activity of the *T. vulgaris* L. essential oil was conducted in Laboratory for Systemic Biomedicine, Division of Molecular Medicine, “Ruđer Bošković” Institute in Zagreb, Croatia. The aim of this study was to evaluate the antiproliferative activity of thyme essential oil by determining the its activity in different concentrations on selected cell lines. For this purpose, three well-characterized cell lines (hematological and solid) were selected according to tumor type, proliferation rate, genotype, growth characteristics and general sensitivity and drug resistance (cell lines used by the National Cancer Institute (NCI) cell line screen). The original substance, as well as stock solution (250 mg/mL/DMSO) were kept in the dark at 4 °C prior to MTT assay. An antitumor drug, doxorubicin, was set up as a positive control (reference compound according to NCI protocol), in parallel with the tested essential oil^44^.

Three human tumor cell lines were used in this study: breast adenocarcinoma (MCF7 ATCC HTB-22), lung carcinoma—large cell lung cancer (NCI-H460 ATCC HTB-177) and acute lymphoblastic leukemia (MOLT-4 ATCC CRL-1582). Cell lines were obtained from American Type Culture Collection (ATCC). All cell lines were routinely cultured as monolayers (MCF-7 and H460) or suspension (MOLT-4) and maintained in RPMI 1640 medium, supplemented with 10% fetal bovine serum (FBS), l-glutamine (2 mM), penicillin (100 U/mL) and streptomycin (100 μg/mL) at 37 °C in a humidified atmosphere with 5% CO_2_^44^.

The effect of the thyme essential oil on the selected cell lines was assessed using the tetrazolium colorimetric MTT assay according to a slightly modified protocol used at the National Cancer Institute^45^. The cells were inoculated at a density of 1.2 × 104 cells/mL (H460), 3 × 104 cells/mL (MCF-7) or 1 × 105 cells/mL (MOLT-4) onto the 96-well microtiter plates on day 0, depending on the doubling times of the specific cell line. Subsequently, six different concentrations (5, 10, 50, 100, 500, and 1000 μg/mL) of the analyzed essential oil were added to the wells, and the plates were incubated for further 72 h. As a control, the reference compound doxorubicin was tested in parallel in five ten-fold dilutions (from 10^–10^ to 10^-6^ M). Working dilutions were prepared on the day of testing. The dimethyl-sulfoxide (DMSO) solvent was also tested for possible inhibitory effect by adjusting its concentration to be the same as in working concentration (concentration of DMSO never exceeded 0.1%). After 72 h of incubation, the cell growth rate was assessed by performing an MTT assay. Next, the essential oil treated medium was discarded and MTT was added to each well at a concentration 20 µg/40 µL and incubated for 4 h. After incubation time, the precipitates were dissolved in 160 μL of DMSO. The absorbance (A) was measured at 570 nm using a microplate reader. The experiment was done in quadruplicate.

A formazan absorbance is in direct correlation with tumor cell viability. The percentage growth (PG) of cell lines was calculated according to the following formulas:

$${\text{If }}\left( {{\text{A test }}{-}{\text{A tzero}}} \right){\text{ }} \ge {\text{ }}0{\text{ then}}:$$

$${\text{PG }} = {\text{ }}\left( {{\text{A test }}{-}{\text{ A tzero}}} \right)/\left( {{\text{A cont }}{-}{\text{ A tzero}}} \right){\text{ }} \times {\text{ 1}}00$$

$${\text{If }}\left( {{\text{A test }}{-}{\text{ A tzero}}} \right){\text{ }} < 0{\text{ then}}:$$

$${\text{PG }} = {\text{ }}\left( {{\text{A test }}{-}{\text{ A tzero}}} \right)/{\text{A tzero}}){\text{ }} \times {\text{ 1}}00,$$

where A tzero is the average absorbance before exposure of cells to the test supstances, A test is the average absorbance after the incubation period (72 h), A cont is the average absorbance after 72 h with no exposure of cells to the test supstances.

### Data analysis

All the experimental results in brine shrimp lethality test (BSLT) were as mean ± SD of three parallel measurements. The percent of mortality (% M) of the brine shrimp nauplii was calculated for each concentration. The median lethal concentration (LC_50_) of the test samples and 95% confidence intervals were determined from the twenty-four hours count using the probit analysis method described by Finney^46^. The LC_50_was derived by linear regression method from plotting probit units against correspondent log of concentration. Probit is a unit of measurement of statistical probability based on deviations from the mean of a normal distribution. The obtained LC_50_ values are compared to Meyer’s and Clarkson’s toxicity criteria^47,48^.

The screening of cytotoxic effects in vitro of the thyme essential oil against tested cell lines (MOLT-4, MCF-7, H460) were estimated in terms of growth inhibition percentage and expressed as IC_50._ 95% confidence intervals were determined. Percent growth inhibition of cells exposed to treatments was calculated as follows:% Inhibition = (100 − PG)/200 × 100*.* Percent of growth inhibition was transformed by probit analysis method described by Finney^46^. The IC_50_was derived by linear regression method from plotting probit units against correspondent log concentration. The criteria used to categorize the cytotoxicity of thyme essential oil based on U.S. National Cancer Institute (NCI) and Geran’s protocol was as follows: IC_50_ ≤ 20 µg/mL = highly cytotoxic, IC_50_ ranged between 21 and 200 µg/mL = moderately cytotoxic, IC_50_ ranged between 201 and 500 µg/mL = weakly cytotoxic and IC_50_ > 501 µg/mL = not cytotoxicity^27^. All the experimental results were as mean ± SD of quadruplicates.

### Ethics declaration

None.

## Supplementary Information

Supplementary Figure S1.

## Data Availability

All data generated/analysed during this study are included in this manuscript (and its Supplementary Information files).
