# Supplementary material for: Cytotoxicity screening of Thymus vulgaris L. essential oil in brine shrimp nauplii and cancer cell lines
Source: Sci Rep. 2021 Jun 23;11:13178. doi: 10.1038/s41598-021-92679-x (PMC8222331; doi:10.1038/s41598-021-92679-x)
Supplement: Supplementary file 1 — Supplementary Figure S1. [file 41598_2021_92679_MOESM1_ESM.pdf]

Cytotoxicity Screening of *Thymus vulgaris* L. Essential Oil in Brine Shrimp Nauplii and Cancer Cell Lines

Haris Nikšić, Fahir Bečić, Emina Korić, Irma Gušić, Elma Omeragić, Samija Muratović, Bojana Miladinović and Kemal Durić

Supplementary Figures:

Supplementary Figure S1. Gas chromatography/mass spectroscopy spectrum of *Thymus vulgaris* L. essential oil native from Bosnia and Herzegovina

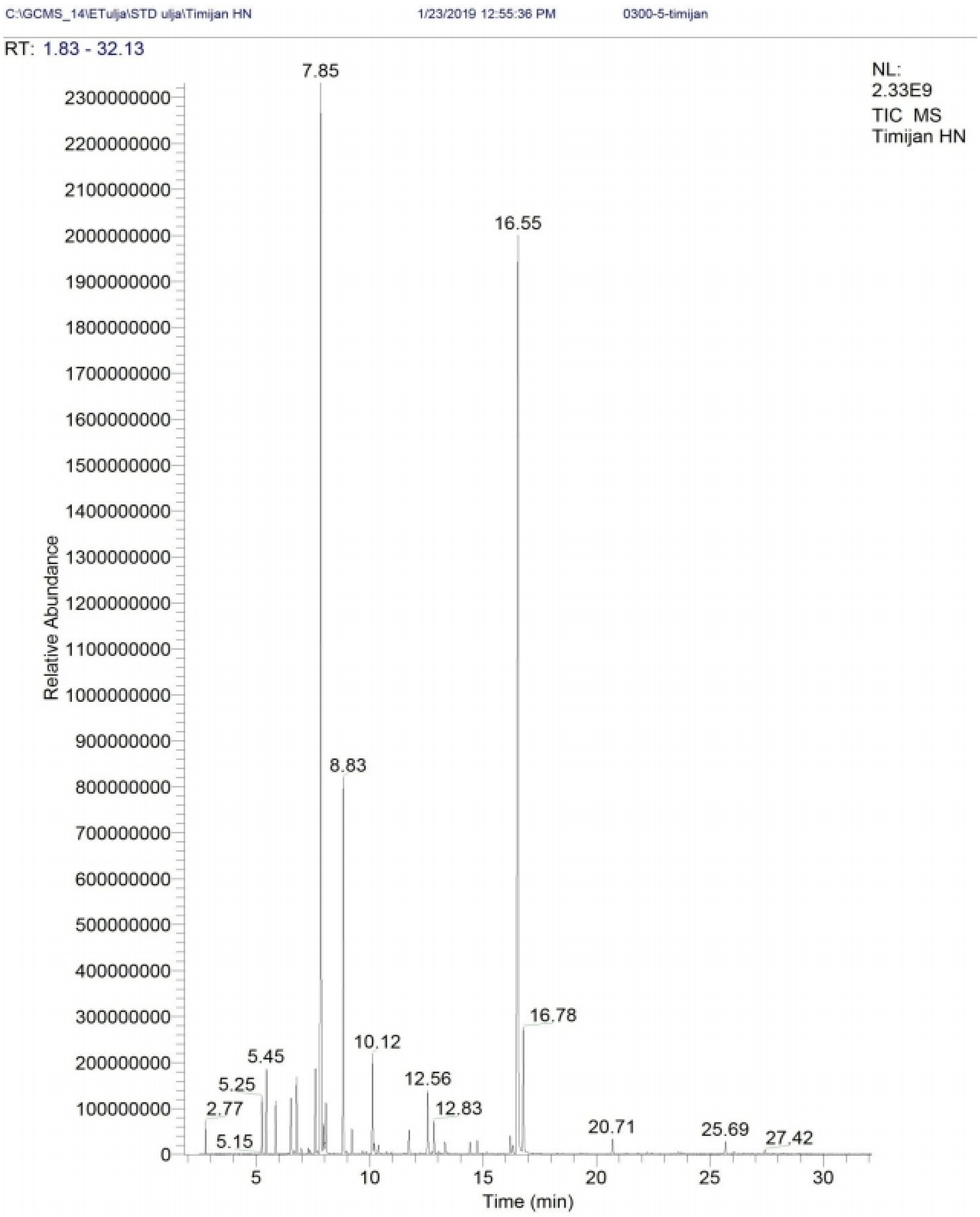

Supplementary Figure S1. Gas chromatography/mass spectroscopy spectrum of *Thymus vulgaris* L. essential oil native from Bosnia and Herzegovina
